# Nitrate of Silver as a Local Application in Erysipelas

**Published:** 1849-08

**Authors:** 


*Nitrate of Silver as a Local Application in Erysipelas, illustrated by two cases.* Read before the District Medical Society for the county of Burlington, April, 1849. By ANDREWS E. BUDD, M. D.

*Case* 1.—On the evening of January 7, 1849, I was called to see a young lady aged about seventeen. She stated that she had been much exposed for three or four days previously, having been sleighing most of the time, and the weather very cold.

Her pulse was rapid, skin dry and hot, tongue coated, and her breathing somewhat oppressed. She complained of pain in her left cheek. On examination, there was discovered a red spot as large as a quarter of a dollar, which was thought to be, by the family, the result of cold, or a chilblain. I stated my fears of approaching erysipelas; still I could not account for the excessive amount of febrile action, and as there was no evidence of inflammation of any organ, I resolved to treat it as a case of fever, and wait the result.

The constitutional symptoms persisted, and the inflammation, which proved to be erysipelas, gradually spread over the left cheek and ear, and over a portion of the left side of the head.

This was the state of things on the fourth day from the attack. The pulse remained pretty steady at 120, though the fever was higher in the afternoon than in the morning. The local applications were in turn, sugar of lead-water, opium water, mercurial ointment, &c., but all did no good. A weak solution of the nitrate of silver was now used, but by the end of the sixth day from the attack, the disease had spread over the head and face completely. The condition of things seemed to be somewhat alarming, and by the advice of Dr. Spencer, the nitrate was made much stronger (vi gr. to 1 oz.) and applied every three hours, over the whole of the face and forehead, and the mercurial ointment to be applied over the head: the patient was directed to take every four hours a teaspoonful of a mixture; containing blue mass xx gr., calcined magnesia 1 dr., water 2 oz. This treatment was continued for four days, when the erysipelas ceased to spread, and the febrile symptoms entirely abated.

The caustic was applied until the soreness disappeared; of course the skin became black after the caustic produced its usual impression. The convalescence was speedy and complete. The skin resumed its healthy appearance in about two weeks from the last application of the remedy.

*Case* 2.—On the morning of the twenty-fourth of March, another case of this most distressing disease presented. The onset was entirely different from the one just under consideration. There had been chilliness alternating with slight flashes of heat, for near a week previous to the appearance of the local disease, want of appetite, headache, &c.

The local affection commenced on the left side of the neck, and in three or four days reached the collar bone below, and spreading upwards covering the ear of the same side. Various local applications were used in turn, but the symptoms gradually increased. There was now much heat of the head, a roaring and ringing sensation in the ears, a heavily coated tongue with red edges, and fever.

Having full confidence in the nitrate of silver, I now commenced its use in the strength of xx gr. to 1 oz., penciling the whole of the diseased surface every three hours, but the erysipelatous inflammation raged violently, and soon covered the whole of the scalp, the opposite ear and side of the neck, forehead and face. The fever raged highest in the morning. The blue mass, magnesia, and water was given every four hours, as an alterative, and with a view also of keeping the bowels open.

Owing to the severity of the disease, it was thought necessary to shear the hair close to the head, that the caustic might be applied over the whole scalp, as well as to the face and neck; in fact it was applied wherever the least trace of the disease could be discovered.

Ice was applied to the head, and cloths dipped in iced water kept constantly upon the forehead. I was extremely fearful that inflammation of the brain would ensue, so great was the heat in the head, and so violent was the fever.

As soon as the caustic had time to cauterize the parts, the soreness and burning subsided, and the fever left as if by magic. Convalescence was speedy.

The cases just described differed materially in the character of the fever. The first commenced with a violent febrile action, and persisted steadily until the cure of the local affection was completed. In this case the fever was equally great when the local inflammation did not cover a space larger than a dollar, as when it covered the whole head and face. In the second there was but little fever at first, but as the local disease progressed, so increased the fever. The fever, too, was of a genuine remittent character. But do not understand me, gentlemen, to suppose that nitrate of silver is applicable to all varieties of erysipelas. We would not recommend it in that form of the disease peculiar to infants, noticed by writers under the name of infantile erysipelas. It is fully described by Drs. Eberle, Condie, and others.

Neither would it be suitable in that variety called Induratio telae cellulosa. I have seen one case of this affection; it occurred in a child two weeks old. A hardened condition of the cuticle, attended with heat, and a slight discoloration of the skin, of a yellowish hue, was discovered near the left breast. This condition of things rapidly spread over nearly the whole body—over one side of the neck, and over one arm. In three or four days from the attack, death came and relieved the little sufferer. The skin could not be pinched up, and the cellular texture seemed hard as stone. There was no change in the parts twelve hours after death; the body was solid, and the arm rigidly fixed as before death.

Gangrenous erysipelas is, I presume, another variety of this affection, not calling for the use of this remedy. But we might, no doubt, use it in phlegmonous erysipelas, and expect as much from its use in arresting the progress of the disease as from any other local application, provided it was commenced early.

The chief object of this paper is to advocate the use of the silver in the treatment of common erysipelas. I believe that it is a safe and efficacious means, and can be relied on as a prompt and almost certain means of cure. It should, no doubt, be used early, and over the whole inflamed surface.

The use of this remedy is not at all new. It has been recommended by most writers on this affection, and many practitioners rely on it as their great means of cure. It should be applied often enough and strong enough to cauterize the parts.

Though many physicians (and I consider myself among the number) rely on this means of treatment, still it must be observed that other means are used by other practitioners, and by them very highly praised.

A solution of the sulphate of iron, formerly more used than now, was thought by some to be almost a specific. At present I believe there is but little confidence in this remedy.

Tincture of iodine and creosote are among the most used at present; the former is recommended by Dr. Wood. He advises that the inflammation should be penciled around its margin, extending over a portion of healthy skin. I presume there could be no impropriety in applying the iodine to the whole surface.

Were I to abandon the nitrate, the next in my esteem would be creosote. This, too, is put on with a camel's hair pencil, and without dilution; covering the whole of the inflamed surface. It is said by those who use it, that it should turn the surface white soon after its application, and that if it fail to do so, the creosote must be considered impure.

Some authors abandon all curative local means, considering the disease destined to run a definite period of time, and recommend that we should conduct the malady safely to its end. Among these is Dr. Watson, of London. He advises only, flannels wrung out of a decoction of poppy-heads, to be applied continually, till relief is had. He states, too, that flour applied by means of a dredging box, afforded much relief to the patients in the hospital, under the care of the apothecary.

But though this idea of assisting to conduct a disease safely to its end, might apply to a whooping cough, or to measles, yet in idiopathic erysipelas, it certainly will not hold good. I feel bound to make every effort to cure a remittent fever, and not wait for the disease to run a certain definite period. I should also feel bound to do all in my power to cure erysipelas, a disease which sometimes hurries its victim off this stage of action in the brief space of three or four days. Wh profess to wield the means of ridding our patients of the poison that may be sapping at their life's blood. These means should enlist our most careful consideration, we should study their adaptation to special and general disease, and thus be ready at all times to alleviate human suffering.

*Medford, April* 16, 1849. *New Jersey Medical Reporter.*

